# The Comprehension of Familiar and Novel Metaphoric Meanings in Schizophrenia: A Pilot Study

**DOI:** 10.3389/fpsyg.2017.02251

**Published:** 2018-01-05

**Authors:** Alexander M. Rapp, Anne K. Felsenheimer, Karin Langohr, Magdalena Klupp

**Affiliations:** Department of Psychiatry and Psychotherapy, University of Tübingen, Tübingen, Germany

**Keywords:** social cognition, figurative language, proverb, schizophrenia, career of metaphor, nonliteral language, semantics, meaningless stimuli

## Abstract

Miscomprehension of nonliteral (“figurative”) language like metaphors, proverbs, idioms, and ironic expressions by patients with schizophrenia is a phenomenon mentioned already in historical psychiatric descriptions. However, it was only recently that studies did differentiate between novel and conventional metaphors, a factor that is known to influence the difficulty of comprehension in healthy subjects. Further, familiarity with stimuli is an important factor for comprehension, which was not recommended in utmost previous studies. In this study, 23 patients with DSM IV schizophrenia and 19 healthy control subjects performed a newly-developed German metaphor comprehension test with three types of stimuli: novel metaphors, conventional German metaphors, and meaningless statements. During the test procedure, participants indicated familiarity with the stimulus and then matched the meaning with one out of four given alternatives. Familiarity rankings did not significantly differ between patients and control subjects. However, on descriptive level, there was a tendency for healthy controls to be more familiar with conventional metaphors than schizophrenic patients. Further, comprehension of conventional and novel metaphors differed significantly between the groups, with higher performance in healthy controls. Considering only those metaphors that had been ranked as familiar, patients only revealed significant lower performance opposed to controls regarding novel metaphors, while they did not differ in conventional metaphors. Taken together, the results indicate that patients with schizophrenia might show an altered way of comprehension in novel metaphors, leading to more misunderstandings. However, their previously reported impairments in conventional metaphors might rather be due to a lack of familiarity with the stimuli—making conventional metaphors to novel metaphors in the individual case.

## Introduction

Figurative language impairment has been documented for a variety of clinical diseases and has fascinated psychiatric researchers and clinicians for decades (Kleist, 1914; Kasanin, 1944; Kanner, 1946). Above that, comprehension and explanation of figurative language are both used to test nonliteral language miscomprehension in psychiatric patients in clinical and research context (Gorham, 1956; Elmore and Gorham, 1957; Rapp and Wild, 2011). Schizophrenia, a serious psychiatric disorder inducing immense personal suffering and economic damage, is an interesting disorder in this context for a number of reasons. The miscomprehension of meanings is a hallmark symptom of schizophrenia. It manifests itself in miscomprehension of intentions, delusionial phenomenae, and language abnormalities (Kleist, 1914; Kasanin, 1944; Crow, 2000; Rapp and Steinhäuser, 2013). Semantic comprehension abnormalities can be severe (Fleischhacker, 1930; DeLisi, 2001), especially for higher order language such as sentences, texts, or nonliteral language (Barrera et al., 2005; Mitchell and Crow, 2005; Li et al., 2009). However, there is conflicting evidence on the extent of the semantic deficit (Moro et al., 2015). Miscomprehension of figurative meanings by schizophrenic patients is a phenomenon already mentioned in historical descriptions (Hadlich, 1931; Goldstein, 1939; Kasanin, 1944; Elmore and Gorham, 1957). The deficit includes all subtypes of figurative language including proverbs (Brattemo, 1962; Rapp et al., 2014), metaphors (Langdon et al., 2002; Schneider et al., 2015; Bambini et al., 2016a), irony (Sparks et al., 2010; Rapp et al., 2013, 2014), and idioms (Titone et al., 2002; Schettino et al., 2010; Sela et al., 2015).

Chapman (Chapman, 1960) was among the first ones to demonstrate experimentally that patients with schizophrenia have abnormalities in metaphor comprehension. In her multiple-choice investigation, subjects matched the meaning of a sentence-level expression (like “David turned yellow when he faced the enemy.”) with a metaphoric (“David became cowardly.”), literal (“David's skin became yellow.”), or a distractor alternative (“David became hungry.”). The main result was that patients with schizophrenia made more errors than controls, both in misinterpreting figurative meanings as literal and vice versa, with the first type of error being significantly more frequent. Chapman‘s finding of altered metaphor comprehension in schizophrenia has been replicated a number of times since then (Rapp and Schmierer, 2010; Zeev-Wolf et al., 2015), including studies of first-episode patients (Anand et al., 1994), remitted subjects (Herold et al., 2002; Mo et al., 2008), and longitudinal studies (Bergemann et al., 2008).

Newer cognitive research on metaphor comprehension in healthy subjects makes it clear that a number of variables influence the difficulty of a metaphor's comprehension process (Schnell, 2007; Gibbs and Colston, 2012; Rapp et al., 2012). For example, cognitive demands vitally differ between easy and difficult metaphors (Coulson and Van Petten, 2002; Glucksberg, 2003; Coulson et al., 2005). As further possible factors, word frequency (Gibbs and Colston, 2012), the culture of the speaker and the recipient (Colston and Katz, 2013), verbal intelligence (Mo et al., 2008), and the context (Giora and Fein, 1999; Mashal and Faust, 2010) are variables that possibly interrelate with how one identifies a correct metaphoric meaning—both in healthy subjects and in patients. A body of literature on healthy and clinical individuals indicates that especially familiarity is acrucial factor to the difficulty of metaphor comprehension (Giora and Fein, 1999; Rapp et al., 2012; Lai et al., 2015).

A widely accepted—but not precisely defined—approach to classify nonliteral language is the distinction between conventional (sometimes called “salient”) metaphors and novel metaphors. A conventional metaphor is “frequently” used in everyday language, whereas a “novel” expression is not.

It is generally accepted that the cognitive processes between conventional and novel metaphors differ (Glucksberg, 2003; Bowdle and Gentner, 2005; Giora, 2007), although the exact nature of these differences is still subject of debate and investigation (Desai et al., 2011; Cardillo et al., 2012). Above that, it is consensus that familiar metaphors are much easier to interpret (Giora, 1999; Colston and Katz, 2013). However, an important remark is that the conventionality of a metaphor in terms of its frequency of use in a language is not inevitably identical with its familiarity in an individual subject. In other words, an individual may be not familiar with even a very “popular,” conventional metaphor. This may sound trivial, but is significant if differences in familiarity are not considered in studies comparing subject populations. In the case of schizophrenia studies, studies that report data for individually perceived familiarity in patients with metaphors are still lacking—even though patients show abnormalities in their everyday use (Kasanin, 1944; Schonauer and Buchkremer, 1986).

Comprehension processes of particularly novel in contrast to conventional metaphors is interesting to analyze in schizophrenia. The mapping of normally unrelated semantic entities is a key process for metaphor comprehension. While some metaphors, like idioms (Cacciari and Tabossi, 1988; Cacciari and Papagno, 2012; Beck and Weber, 2016), may be processed as fixed expressions and less rely on these mapping processes, this is impossible for novel metaphors. For example, in the case of the “Neuroimaging is a gold mine” metaphor, it is necessary to map the analogy between the entities of imaging research and mining (Glucksberg, 2003). There is good imaging and brain lesion evidence that the left lateral inferior frontal gyrus is a key region for this process (Rapp et al., 2004, 2011, 2012), a brain region which is structurally and functionally abnormal in schizophrenia (Heckers, 1997; Chan et al., 2011; Rapp and Steinhäuser, 2013). Indeed, patients with schizophrenia show fMRI activation abnormalities in this brain region during comprehension of novel metaphors (Kircher et al., 2007; Mashal et al., 2013; Schneider et al., 2015), which even correlate with their severity of concretism (Kircher et al., 2007). In an fMRI study from our group (Kircher et al., 2007), patients with schizophrenia showed borderline significance in their impairment in a semantic connotation task with novel metaphors. Using the same metaphors but more subjects, this group effect was clearly significant in another study (Schneider et al., 2015). In a seminal fMRI study in Hebrew language, Mashal et al. (2014) investigated 14 patients with schizophrenia and 14 matched healthy controls. Subjects evaluated word pairs which were either conventional metaphoric related, novel metaphoric, literal, or unrelated in their meanings and judged the meaningfulness of the word pairs. Results indicated a significant difference between patients and controls for novel and conventional metaphors, while performance for unrelated word pairs was not significantly impaired. However, in another Hebrew study, Zeev-Wolf et al. (2014) investigated metaphor comprehension in 17 individuals with schizophrenia and 30 matched controls with a contradictory result. Similar to Mashal et al. (2014), novel metaphoric, conventional metaphoric, literal, and unrelated word pairs were used as stimuli and participants indicated the meaningfulness of the stimuli by pressing one out of two buttons with their right index finger. While error rates for conventional metaphors were low compared to the literal stimuli, there were even slightly better performance with novel metaphors than controls. Schizophrenia patients showed a drastic higher error rate for unrelated word pairs. Another German language study with novel and conventional metaphors is published by Mossaheb et al. (2014). In their study, 40 patients with schizophrenia-spectrum disorders and 43 healthy control subjects were investigated in their comprehension for novel and conventional metaphors. In both tests, schizophrenia patients showed impairment, however, with a more pronounced difference for conventional metaphors. In a study in Bengali, Chakrabarty et al. (2014) found decreased performance for both conventional and novel metaphors in patients with schizophrenia. In a study with 19 schizophrenia patients and 19 control subjects, Varga et al. (2014) investigated the comprehension of novel and conventional Hungarian metaphors using a verbal explanation task (Drury et al., 1998) and found a possible role of IQ to associate with comprehension. There was no significant difference between patients and controls for conventional metaphors, however, a significant difference in performance was detectable for unconventional Hungarian metaphors. To note, no study using the English language novel metaphors is available for schizophrenia.

It is usually assumed that patients with schizophrenia show a tendency toward literal misinterpretation of metaphoric meanings. However, even Chapman's (Chapman, 1960) historical investigation highlights that the error pattern in schizophrenia also includes the opposite error, which is the metaphoric interpretation of literal-intended sentences. This type of error, which is also frequently seen for other nonliteral language like proverbs and irony (Hensler, 2009; Rapp et al., 2014), may represent a correlation of widening semantic associations in some patients with schizophrenia (Kircher, 2003; Kircher et al., 2007; Zeev-Wolf et al., 2015). Wide semantic associations, meaning the “openness” of the recipient to accept semantic interrelations at the “borderline” would theoretically facilitate acceptance of novel metaphoric relationships on the one hand (Faust and Weisper, 2000; Rapp et al., 2004; Mashal et al., 2005) and is a well-known phenomenon in thought-disordered patients with schizophrenia on the other hand (Spitzer et al., 1993; Kircher et al., 2001, 2007). Both an impaired and facilitated performance in novel metaphor comprehension would therefore make sense in schizophrenia. Of note here Zeev-Wolf et al. (2014) reported increased performance of schizophrenic patients in a novel comprehension task relative to control subjects.

In this pilot study, we therefore aim to investigate comprehension of conventional metaphors, novel metaphors and meaningless statements in one paradigm in schizophrenia. Our hypothesis is that in a multiple-choice format, patients with schizophrenia will show both an elevated miscomprehension of metaphoric sentences as literal and an elevated miscomprehension of meaningless statements as metaphorical-intended. However, as the conventionality of a metaphor in general might not reflect the familiarity with the metaphor in an individual, we further address if possible differences still remain when only perceived familiar metaphors are compared between healthy adults and schizophrenia patients.

## Materials

### Subjects

The study was approved by the local ethical committee (University of Tübingen, Germany). Twenty three patients (15 females) with DSM IV schizophrenia and 19 healthy control (HC) subjects (9 females) gave written informed consent and participated in the study. All participants were German native speakers. Patients were recruited from the Department of Psychiatry and Psychotherapy at the University Hospital Tübingen, Germany. Sixteen were inpatients and six outpatients. Mean duration of illness was 10.5 years (SD: 7.8). All patients were on stable medication, mainly with atypical antipsychotics [mean dose 365 chlorpromazine equivalents (SD: 255; Andreasen et al., 2010)]. Control subjects were recruited from the general population and were free from psychiatric illness. There were no significant differences in gender [*t*_(39)_ = −1.35, *p* = 0.186], age [*t*_(39)_ = 1.99, *p* = 0.053] and educational achievement [*t*_(38.59)_ = −1.63, *p* = 0.126] between the groups. Mean age was 42.3 years (range 24–62) in the patient group and 34.0 years (range 21–62) in the control group.

### Metaphor comprehension test

A German language metaphor comprehension test was developed *de novo*. The test consists of 39 items (Appendix 1). There are three types of stimuli: novel metaphors (like “a tender sting”), conventional metaphors (like “break a heart”) and meaningless statements (like “sport of citrons”). Conventional metaphors originate from everyday German language, while novel metaphors and meaningless statements were created *de novo*. During test development, all stimuli and additionally their slight modifications were cross-checked for occurrence in the “Google” corpus. All conventional metaphors showed high occurrence in Google, whereas novel metaphors and meaningless statements showed no or extremely low (<10 hits) occurrence.

During the test procedure, the participant first indicates if he/she is familiar or unfamiliar with the phrasing or figure of speech. Then, the subject must match the meaning with one out of four given alternatives: one depicting the (correct) metaphorical meaning, a distractor describing the literal meaning, a distractor with an unrelated meaning or the selection “this phrase does not make sense.” For meaningless statements, the latter represents the correct answer, whereas for the other stimuli the metaphorical meaning is rated as correct. There are 13 stimuli for each stimulus type. Each correct answer counts as one point.

### Procedure

First, all subjects received complete information about the study and ability to consent was ensured. Then, participants were given both written and oral information about the test and completed a practice session with two stimuli not used in the experiment. Subjects then completed the metaphor comprehension test. If requested by the subject, a short break during the test procedure was possible. SPSS 24 was used for statistical analyses.

## Results

### Statistical analysis

Repeated measure analysis of variance (rmANOVA) with group (schizophrenic patients vs. healthy controls) as between-subjects factor, the stimulus type (novel metaphor, conventional metaphor, meaningless utterances) as within-subjects factor and either familiarity ranking or accuracy rates as dependent variable were conducted. Due to the small sample size and the exploratory character of the study Alpha was set at 0.05, two tailed. In case of unequal variances indicated by Levene's test, Welch test was calculated and Bonferroni correction applied for *post-hoc* comparison of group within stimulus types. Above that, Cohen's d was calculated for each of these group comparisons, with a small effect size indicated by *d* = 0.2, a medium by *d* = 0.5 and a large by *d* = 0.8 (Cohen, 1988).

### Familiarity with the stimuli

During the test procedure, subjects classified each stimulus as being “familiar” or “unfamiliar.” To examine the individually perceived familiarity between groups and every type of nonliteral language, rmANOVA was applied with group (patients vs. HC) as between-subjects factor, metaphor type (conventional, novel, meaningless) as within-subjects factor and familiarity scores as dependent variable. Level of significance was set to *p* < 0.05. Since Mauchly's test of sphericity was significant, Greenhouse-Geiser correction was applied. Results revealed a significant main effect for condition [Greenhouse-Geisser *F*_(1.48, 59.19)_ = 368.89, *p* < 0.001, partial η^2^ = 0.90] and group by familiarity interaction [Greenhouse-Geisser *F*_(1.48, 59.19)_ = 4.88, *p* < 0.019, partial η^2^ = 0.11]. No statistically significant main effect for group was found [*F*_(1, 40)_ = 0.02, *p* = 0.888, partial η^2^ = 0.00]. *Post-hoc* pairwise comparison using the Bonferroni correction exploring the main effect of stimulus type revealed significant difference in familiarity ranking between all conditions (all *p* < 0.001), confirming that conventional metaphors (*M* = 10.83) in the test are perceived as more familiar than novel metaphors (*M* = 2.46) and meaningless utterances (*M* = 0.6). To explore the interaction of group by condition, one-way ANOVA was conducted. However, only a trend for higher familiarity ratings for conventional metaphors in control subjects compared to patients [Welch's *F*_(1, 28.59)_ = 4.19, *p* = 0.05], but no statistically significant differences for novel metaphors [Welch's *F*_(1, 37.60)_ = 0.34, *p* = 0.57] and meaningless utterances [Welch's *F*_(1, 24.20)_ = 2.84, *p* = 0.105]. Table 1 shows means, standard deviations and Cohen's d for the familiarity rating.

**Table 1:** Mean values, standard deviations and cohen's d for healthy controls (HC) and schizophrenia patients (SP) in rankings of perceived familiarity.

**Stimulus type**	**HC (*****n***** = 19)**		**SP (*****n***** = 23)**		**Cohen's d (HC > SP)**
	**Mean**	**SD**	**Mean**	**SD**	
Novel metaphors	2.3	1.6	2.7	2.6	−0.19^+^
Conventional metaphors	11.6	1.2	10.1	3.2	0.62 (n.s.)
Meaningless stimuli	0.2	0.5	1.0	2.5	−0.44 (n.s.)

*Each familiar stimulus is counted with a score of one, thus the highest reachable score for each subgroup would be 13. rmANOVA shows a statistically significant main effect for stimulus type, confirming that conventional metaphors are seen much more familiar than novel metaphors and meaningless utterances*.

+ * = p < 0.1*.

### Comprehension of metaphor types

**Figure 1:**
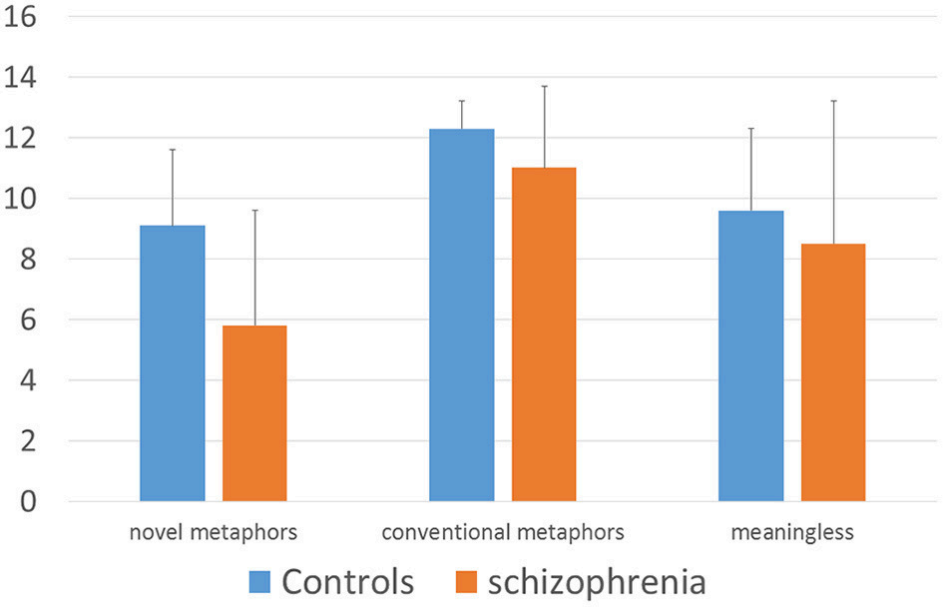
Performance of patients with schizophrenia (*n* = 23) and healthy control subjects (*n* = 19) in a new developed German multiple choice metaphor comprehension test. Mean performance and standard deviations. A value of 13 would indicate perect performance.

To test the hypothesis of performance in metaphor comprehension depending on type of nonliteral language, another rmANOVA was calculated, entering group (patients vs. controls) as between-subjects factor, metaphor type (conventional, novel, meaningless) as within-subjects factor and accuracy rates as dependent variable. Again, level of significance was set to *p* < 0.05 and Greenhouse-Geiser correction was applied, when Mauchly's test was significant. Results showed significant main effects for metaphor type [Greenhouse-Geisser *F*_(1.34, 53.53)_ = 19.04, *p* < 0.001, partial η^2^ = 0.32] and group on accuracy rates [*F*_(1, 40)_ = 9, 97, *p* = 0.003, partial η^2^ = 0.20] (Figure 1). There was no significant interaction between metaphor type and group [Greenhouse-Geisser *F*_(1.34, 53.53)_ = 1.62, *p* = 0.211, partial η^2^ = 0.04]. One-way ANOVA was conducted to assess the specific effect of group. Accuracy rates differed statistically significant on conventional metaphors [Welch's *F*_(1, 28.09)_ = 4.69, *p* = 0.039] and novel metaphors [Welch's *F*_(1, 38.08)_ = 11.63, *p* = 0.002], but not on meaningless utterances [Welch's *F*_(1, 35.86)_ = 0.91, *p* = 0.347], with schizophrenics revealing less correct responses than controls in novel (*M* = 5.78 vs. *M* = 9.1) and conventional metaphors (*M* = 10.96 vs. *M* = 12.26). *Post-hoc* pairwise comparisons using the Bonferroni correction exploring the main effect of metaphor type revealed significant difference in correct responses between conventional metaphors and meaningless utterances (*p* = 0.001), conventional metaphors and novel metaphors (*p* < 0.001), indicating higher accuracy scores in conventional (*M* = 11.55) than in novel metaphors (*M* = 9.02) and meaningless utterances (*M* = 7.29). Differences between novel metaphors and meaningless utterances were not significant (*p* = 0.208). Table 2 reports means, standard deviations, and cohen's d for every language condition.

**Table 2:** Performance in the multiple choice test.

**Stimulus type**	**HC (*****n***** = 19)**		**SP (*****n***** = 23)**		**Effect size (Cohen's d) HC > SP**
	**Mean**	**SD**	**Mean**	**SD**	
Novel metaphors	9.1	2.5	5.8	3.8	0.29^**^
Conventional metaphors	12.3	0.9	11.0	2.7	0.65^*^
Meaningless stimuli	9.6	2.7	8.5	4.7	1.03 (n.s.)

*Mean values, standard deviations and cohen's d for healthy controls (HC) and schizophrenia patients (SP) in metaphor types. A mean value of 13.0 would indicate perfect performance in any subject. The rmANOVA resulted in significant main effects of metaphor type and group, but no significant interaction. n.s. = not statistically significant*,

* *p < 0.05*,

** *p < 0.01, Post-hoc Welch-Test*.

### Comprehension of familiar metaphors

In a last step, we aimed to investigate if the previously revealed lower accuracy rates in schizophrenia patients in conventional and novel metaphors might be due to a lack of familiarity with the stimuli (Table 3). Therefore, another rmANOVA with group (patients vs. controls) as between-subjects factor and metaphor type (novel vs. conventional) as within-subject factor was conducted. As dependent variable we selected the proportion of correct identified metaphors that had been selected as being familiar to the participant divided by the total number of familiar stimuli in each condition. Results showed significant main effects for metaphor type [Greenhouse-Geisser *F*_(1, 40)_ = 19.73, *p* < 0.001, partial η^2^ = 0.33] and group on accuracy rates [*F*_(1, 40)_ = 9.24, *p* = 0.004, partial η^2^ = 0.19], as well as a significant interaction between metaphor type and group [Greenhouse-Geisser *F*_(1, 40)_ = 5.38, *p* = 0.026, partial η^2^ = 0.12]. Again, *post-hoc* pairwise comparisons using the Bonferroni correction for the main effect of metaphor type revealed significant difference in correct responses between conventional metaphors and novel metaphors (*p* < 0.001), indicating higher proportion of correct responses in familiar conventional metaphors (*M* = 0.95) than in familiar novel metaphors (*M* = 0.67). Further, one-way ANOVA to explore the interaction of group and metaphor type revealed statistically significant differences in familiar novel metaphors between patients and controls [Welch's *F*_(1, 38.34)_ = 8.48, *p* = 0.006], with schizophrenic patients being less accurate than controls (*M* = 0.51 vs. *M* = 0.86). However, groups did not differ in the proportion of correct responses of familiar conventional metaphors [Welch's *F*_(1, 23.44)_ = 2.69, *p* = 0.115].

**Table 3:** Performance in individually familiar perceived metaphors.

**Stimulus type**	**HC (*****n***** = 19)**		**SP (*****n***** = 23)**		**Effect size (Cohen's d) HC > SP**
	**Mean**	**SD**	**Mean**	**SD**	
Familiar novel metaphors	0.86	0.32	0.51	0.45	0.9^*^
Familiar conventional metaphors	0.99	0.03	0.93	0.17	0.49 n.s.

*Mean values, standard deviations and cohen's d for healthy controls (HC) and schizophrenia patients (SP) in metaphor types. Scores were calculated as the amount of correct responses divided by the amount of familiar ranked metaphors each. The rmANOVA resulted in significant main effects of metaphor type and group and a significant interaction. n.s. = not statistically significant*,

* *p < 0.05, Post-hoc Welch-Test*.

## Discussion

We investigated the comprehension of metaphors and meaningless sentences using a new developed German multiple choice test containing conventional metaphors, novel metaphors, and meaningless expressions (Appendix 1), In the test, the stimuli are first classified as either familiar or nonfamiliar. Afterwards, stimuli must be matched with one out of four multiple-choice alternatives: a description of the metaphoric, literal, and unrelated meaning or the classification “not understandable.” The use of a multiple-choice approach has the advantage of an easy and precise score. However, it represents a cognitive operation that is different from the verbal explanation procedure which is mostly applied in clinical routine (Winner and Gardner, 1977; Rapp, 2009). Brain lesion research indicates that the multiple choice approach has a higher right cerebral hemisphere processing involvement in comparison to verbal explanation (Winner and Gardner, 1977; Gagnon et al., 2003; Rapp et al., 2012), possibly due to the fact that false alternatives need to be inhibited in order to select the correct one.

Our test is supplemental to existing figurative language paradigms in German language (Kogan and Chadrow, 1986; Barth and Kufferle, 2001; Uekermann et al., 2008; Mossaheb et al., 2014). Since perceived familiarity for conventional was higher than for novel metaphors and meaningless utterances in this study, results confirmed our newly constructed stimulus pools for each category.

Integrating the aspect of subjective familiarity with the presented metaphor, the test contributed to previous tasks using nonliteral language in general, or metaphors specifically. Like our test, the University of Münster proverb test (Uekermann et al., 2008; Thoma et al., 2009) directly addresses familiarity with the stimuli. However, even though they addressed the nonliteral figure of speech proverbs, they neither included novel nor meaningless stimuli. Compared to that, those tests that do include metaphors specifically—like the Austrian tests by Barth (Barth and Kufferle, 2001) and Mossaheb (Mossaheb et al., 2014)—do not assess familiarity directly and consist of conventional expressions only. With regard to familiarity, the same goes for the metaphor triad test by Kogan and Chadrow (1986), in which the subject has to identify novel metaphorical relationships between given alternatives.

The main result of this study is that patients with schizophrenia showed a significantly lower overall performance than matched healthy controls in the comprehension of metaphors in our multiple-choice test. Contrary to our expectations, patients did not differ from controls in performance on meaningless utterances, implying no elevated misunderstanding of non-metaphoric stimuli as metaphors. Although results showed that novel metaphors are more complicated to comprehend than conventional metaphors in both groups, patients still had more difficulties in interpreting novel and conventional metaphors than healthy controls. This does not only contribute to the assumption that conventional and novel metaphors depend on different cognitive processes, but is also in line with previous research indicating abnormalities in figurative language processing in schizophrenia (Gorham, 1956; Andreasen, 1977; Papagno, this issue, Iakimova et al., this issue). However, perhaps the most interesting finding of the study was that the lower performance of patients in the comprehension of conventional metaphors disappeared when only those metaphors were taken into account, that had been ranked as familiar by the individual. This further strengthens our advice to ensure familiarity with the individual stimulus when metaphor comprehension is used in a psychiatric assessment (Rapp and Wild, 2011). Further, it decrements a research gap for comprehension of metaphors specifically in schizophrenia by highlighting the importance of differentiating the conventionality of a metaphor on a general and an individual level. Done otherwise, it might be leading to alleged impairments in metaphor comprehension in patients, that are rather be due to a lack of knowledge. Hereby, some metaphors categorized as conventional might rather be seen as novel ones, which were even for healthy individuals more difficult to comprehend than conventional ones. However, patients still had more problems than controls in the comprehension of novel metaphors, that had been perceived as familiar—indicating that, nonetheless, there might be a different way of comprehension processes in patients with schizophrenia leading to more misinterpretations.

All these investigations indicate that metaphor comprehension deficits in schizophrenia are better classified as a difficulty rather than an “inability” (Epelbaum et al., 1992; de Bonis et al., 1997; Drury et al., 1998; Langdon et al., 2002; Hensler, 2009; Papagno, this issue) assumed in historical descriptions (Finckh, 1906; Storch, 1922). We conclude from our results our test is suitable for testing figurative language impairment in schizophrenia and, possibly, other clinical conditions. The majority of clinical metaphor research has been focused on autism and schizophrenia, but some studies have investigated other clinical populations including dementias (Rapp and Wild, 2011), William's syndrome (Annaz et al., 2009), depression (Iakimova et al., 2006), traumatic brain injury (Martin and McDonald, 2005), relational aggression (Blasko and Kazmerski, 2006), schizotypal personality traits (Humphrey et al., 2010; Ettinger et al., 2015), Parkinson's disease (Gutmann, 2009), amyotrophic lateral sclerosis (Bambini et al., 2016b), and other developmental disorders (Rapp and Wild, 2011). Paradigms on metaphor and idiom comprehension are also used as clinical and research tool to investigate embodiment (Gibbs et al., 2004; Denke et al., 2014) and motor language (Raposo et al., 2009), social cognition (Langdon et al., 2002; Landau et al., 2010), aphasia (Papagno and Caporali, 2006), intelligence (Jäger and Althoff, 1994), and coverbal gestures (Straube et al., 2014).

### Familiarity with metaphors in schizophrenia

Research from healthy and brain damaged subjects indicates that the comprehension process of a metaphor process varies in relation to its conventionality and familiarity (Desai et al., 2011; Lai et al., 2015). Familiarity with a metaphor likewise facilitates its comprehension process (Bowdle and Gentner, 2005; Schnell, 2007). A significant difference in familiarity with the stimuli can therefore represent a confounding factor in comparisons of clinical and non-clinical populations. Astonishingly, research in schizophrenia so far did not address this aspect specifically. However, alike in clinical context (Rapp and Wild, 2011), we would recommend to test familiarity with the individual figurative stimuli in future research. In our study with a predominantly chronic sample, on the descriptive level patients showed a tendency to be less familiar with conventional metaphors than controls, but did not differ significantly in their perceived familiarity of meaningless utterances and novel metaphors. This finding is partly in contrast to findings for other types of figurative language. For example Thoma et al. (2009) found significant differences in familiarity with German proverbs in patients with schizophrenia. An explanation for this difference not reaching significance in the current study could be that their test used a 5-point likert scale to assess familiarity, contrary to a dichotomous classification here. Research using more sophisticated methods of familiarity and sentence level stimuli including other types of figurative language seems eligible.

### Novel metaphor comprehension in schizophrenia

Our test enables to analyse comprehension of novel metaphors specifically. Both an impairment, due to generally impaired language skills (Mitchell and Crow, 2005) and facilitated performance due to increased ability to establish semantic associations (Spitzer et al., 1993), for novel metaphors would be compatible with previous research. Our pilot study strengthens findings from the majority of previous studies as it indicates an impairment rather than a facilitated performance. While our significant group difference for novel metaphors demonstrates a present potential of our test to graph novel metaphor comprehension in a clinical population, it is clear for a long time that seeing schizophrenias as a homogeneous group represents a severe simplification of schizophrenia psychopathology. The psychopathology of schizophrenia is not at all homogeneous. The comprehension of figurative language is not stable over time in schizophrenia (Braff et al., 1988; Drury et al., 1998; Rapp et al., 2007) and differs dramatically between individuals. It is likely that a number of factors is associated with metaphor comprehension in schizophrenia. Especially thought disorder is an important candidate among these factors since both thought disorder and novel metaphor comprehension directly relate to semantic mapping (Spitzer et al., 1994; Kircher et al., 2001, 2007; Zeev-Wolf et al., 2014). Theoretically, the impact of thought disorder on metaphor comprehension might be elevated for novel metaphors in comparison with familiar ones as their demand to establish novel semantic relationships is higher.

## Limitations and implications

We are aware of several limitations in our study. Our binary classification of each stimulus as “familiar” vs. “unfamiliar” simplifies procedure for the study subjects and data analysis and was chosen here to enhance clinical applicableness of our test. However, research specifically addressing this aspect indicates metaphor familiarity may represent a dimensional phenomenon (Blasko and Connine, 1993; Lai et al., 2015). Theoretically, differences in age between patients and controls—although not significant—could have influenced our results.

An important limitation relates to the generalizability of the results. In this pilot study, we investigated a group of mainly subacute schizophrenia, in- and outpatients, without further specifying a subtype or psychopathology. It must be strengthened again that other suggested associated variables for nonliteral language comprehensions in schizophrenia are in discussion. These include IQ (Brüne and Bodenstein, 2005; Varga et al., 2014), duration of illness (Anand et al., 1994), chronicity (Watson, 1976), positive symptoms (Drury et al., 1998), schizotypy (Langdon and Coltheart, 2004; Humphrey et al., 2010; Rapp et al., 2010, 2013; Ettinger et al., 2015), medication (Levy, 1968; Krystal et al., 1998), subtype (Watson, 1976; de Bonis et al., 1997), cognitive deficits (Mo et al., 2008), delusions (Drury et al., 1998; Rhodes and Jakes, 2004), and other psychopathology. While we conclude our metaphor test is a suitable tool to investigate interaction with these factors, we did not yet recommend them in this study.

Another more general issue, perhaps, is the point of what represents an error in our test and what does not. In our study, any answer given in accordance to the test developers assumption are scored as “correct.” This approach seems reasonable for meaningless statements (with their extremely low familiarity rates) and conventional metaphors (with checked high prevalences in the google corpus). However, in the case of novel metaphoric relationships, the decision that “this phrase does not make sense” may represent a “willingness” to accept meanings within the range of normality rather than a pathology or cognitive deficit. Future research may clarify how novel metaphor acceptance relates to creativity on one (Humphrey et al., 2010 Kennett and Faust, this issue) and delusion proneness (Nunn and Peters, 2001; Langdon and Coltheart, 2004; Rapp et al., 2010, 2014) on the other hand.

## Ethics statement

This study was carried out in accordance with the recommendations of “name of guidelines, name of committee” with written informed consent from all subjects. All subjects gave written informed consent in accordance with the Declaration of Helsinki. The protocol was approved by the Ethical committee of the University of Tübingen.

## Author contributions

AR: designed the study. KL and MK: collected the data. MK, AF, and AR: did the analysis. AF and AR: wrote the manuscript.

### Conflict of interest statement

The authors declare that the research was conducted in the absence of any commercial or financial relationships that could be construed as a potential conflict of interest.

## Appendix

The following stimulus pool of the Tübinger Metaphern Test is translated from German language.

**Table:**

**Stimulus**	**Type**
1. “Television tower” (Fernsehturm)	*Building*
2. “Snow gently trickles” (leise rieselt der Schnee)	*German christmas carol*
3. “Small scrumb of love” (ein Krümelchen Liebe)	(NM1)
4. “Fruit juice banishment” (Fruchtsaftverbannung)	(M1)
5. “Water under the bridge” (Schnee von gestern)	(CM1)
6. “Stand a round” (eine Runde schmeißen)	(CM2)
7. “To spurn the law” (Recht mit Füßen treten)	(CM3)
8. “Lascivious bus station” (lüsterne Bushaltestelle)	(M2)
9. “Overheated decision” (überhitzte Ehescheidung)	(NM2)
10. “Carry someone under the heart” (Jemanden unter dem Herzen tragen)	(CM4)
11. “Translucent moment” (lichtdurchlässiger Augenblick)	(NM3)
12. “Hit the nail on the hand” (Nagel auf den Kopf treffen)	(CM5)
13. “To abet someone” (etwas Vorschub leisten; no correct translation found)	(CM6)
14. “To be well oiled” (einen auf die Lampe gießen)	(CM7)
15. “Break a heart” (Jemandem das Herz brechen)	(CM8)
16. “A Porsche in love” (ein verliebter Porsche)	(M3)
17. “Dried ski-jumping” (getrocknetes Skispringen)	(M4)
18. “Dried violins” (getrocknete Violinen)	(M5)
19. “Retired railway” (berentete Lokomotive)	(NM4)
20. “To give ear to someone (Jemandem ein Ohr schenken)	(CM9)
21. “A borrowed Beauty” (eine geliehene Schönheit)	(NM5)
22. “Sport of citrons” (Zitronen-Sport)	(M6)
23. “Chocolaty relationship” (schokoladige Beziehung)	(NM6)
24. “Swing of widows” (Witwenschaukel)	(NM7)
25. “Rainy civil service” (eine verregnete Behörde)	(M7)
26. “To have money to burn” (Geld wie Heu haben)	(CM10)
27. “Sober alcoholic” (trockener Alkoholiker; no correct translation found)	(CM11)
28. “Athletic chair” (sportlicher Stuhl)	(M8)
29. “Stroke puzzle” (Schlaganfall-Rätsel)	(M9)
30. “Wall of Silence” (Mauer des Schweigens)	(CM12)
31. “By clean feet” (Sauberen Fußes)	(NM8)
32. “Convenience locker” (Bequemlichkeits-Schließfach)	(M10)
33. “A tender sting (ein zarter Stachel)	(NM9)
34. “Patient horror” (geduldiger Schrecken)	(M11)
35. “Sugared hospital” (gezuckertes Krankenhaus)	(M12)
36. “Boozy all-terrain vehicle” (versoffener Geländewagen)	(NM10)
37. “Immature desire” (unreifes Verlangen)	(NM11)
38. “Flooding lust” (überflutende Lust)	(NM12)
39. “Oblique boon of the home” (schiefer Haussegen; no correct translation found)	(CM13)
40. “Snowy apple pie” (verschneiter Apfelkuchen)	(NM13)
41. “Paragraph drum” (Absatztrommel)	(M13)

*As far as possible, English counterparts for conventional metaphors were used. Stimulus material is built upon three types of nonliteral language comprising 13 items each: novel metaphor (NM) conventional metaphor (CM) meaningless utterances (M). First two items of the test are only for practice reasons and do not count into total scores. Every item must be rated with regard to familiarity (yes/no). The meaning of every items is evaluated via four multiple choice options comprising the (correct) metaphorical meaning, two distractors describing the literal and an unrelated meaning and the selection “this phrase does not make sense.” For meaningless statements, the latter represents the correct answer, whereas for the other stimuli the metaphorical meaning is rated as correct. Each correct answer counts as 1 point. Correct identifications are scored one resulting in a total sumscore for metaphor comprehension and three subscores for every type of nonliteral language*.

## References

[B1] Anand A. Wales R. J. Jackson H. J. Copolov D. L. (1994). Linguistic impairment in early psychosis. J. Nerv. Ment. Dis. 182, 488–493. 10.1097/00005053-199409000-00002 8083676

[B2] Andreasen N. C. (1977). Reliability and validity of proverb interpretation to assess mental status. Compr. Psychiatry 18, 465–472. 10.1016/0010-440X(77)90046-3 891169

[B3] Andreasen N. C. Pressler M. Nopoulos P. Miller D. Ho B. C. (2010). Antipsychotic dose equivalents and dose-years: a standardized method for comparing exposure to different drugs. Biol. Psychiatry 67, 255–262. 10.1016/j.biopsych.2009.08.040 19897178 PMC3677042

[B4] Annaz D. Van Herwegen J. Thomas M. Fishman R. Karmiloff-Smith A. Rundblad G. (2009). Comprehension of metaphor and metonymy in children with Williams syndrome. Int. J. Lang. Commun. Disord. 44, 962–978. 10.1080/13682820802525005 19874091

[B5] Bambini V. Arcara G. Bechi M. Buonocore M. Cavallaro R. Bosia M. (2016a). The communicative impairment as a core feature of schizophrenia: frequency of pragmatic deficit, cognitive substrates, and relation with quality of life. Compr. Psychiatry 71, 106–120. 10.1016/j.comppsych.2016.08.012 27653782

[B6] Bambini V. Arcara G. Martinelli I. Bernini S. Alvisi E. Moro A.. (2016b). Communication and pragmatic breakdowns in amyotrophic lateral sclerosis patients. Brain Lang. 153, 1–12. 10.1016/j.bandl.2015.12.002 26799425

[B7] Barrera A. McKenna P. J. Berrios G. E. (2005). Formal thought disorder in schizophrenia: an executive or a semantic deficit? Psychol. Med. 35, 121–132. 10.1017/S003329170400279X 15842035

[B8] Barth A. Kufferle B. (2001). Die Entwicklung eines Sprichworttests zur Erfassung konkretistischer Denkstörungen bei schizophrenen Patienten. Nervenarzt 72, 853–858. 10.1007/s001150170019 11758092

[B9] Beck S. D. Weber A. (2016). Bilingual and monolingual idiom processing is cut from the same cloth: the role of the L1 in literal and figurative meaning activation. Front. Psychol. 7:1350. 10.3389/fpsyg.2016.01350 27667979 PMC5016721

[B10] Bergemann N. Parzer P. Jaggy S. Auler B. Mundt C. Maier-Braunleder S. (2008). Estrogen and comprehension of metaphoric speech in women suffering from schizophrenia: results of a double-blind, placebo-controlled trial. Schizophr. Bull. 34, 1172–1181. 10.1093/schbul/sbm138 18156639 PMC2632488

[B11] Blasko D. G. Connine C. M. (1993). Effects of familiarity and aptness on metaphor processing. J. Exp. Psychol. Learn. Mem. Cogn. 19, 295–308. 10.1037/0278-7393.19.2.295 7681095

[B12] Blasko D. G. Kazmerski V. A. (2006). ERP correlates of individual differences in the comprehension of nonliteral language. Metaphor Symbol 21, 267–284. 10.1207/s15327868ms2104_4

[B13] Bowdle B. F. Gentner D. (2005). The career of metaphor. Psychol. Rev. 112, 193–216. 10.1037/0033-295X.112.1.193 15631593

[B14] Braff D. L. Glick I. D. Johnson M. H. Zisook S. (1988). The clinical-significance of thought-disorder across time in psychiatric-patients. J. Nerv. Mental Dis. 176, 213–220. 10.1097/00005053-198804000-00004 3351500

[B15] Brattemo C.-E. (1962). Interpretations of proverbs in schizophrenic patients. Further studies. Acta Psychol. 20, 254–263. 10.1016/0001-6918(62)90022-7

[B16] Brüne M. Bodenstein L. (2005). Proverb comprehension reconsidered—'theory of mind'and the pragmatic use of language in schizophrenia. Schizophr. Res. 75, 233–239. 10.1016/j.schres.2004.11.006 15885515

[B17] Cacciari C. Papagno C. (2012). Neuropsychological and neurophysiological correlates of idiom understanding: how many hemispheres are involved?, in The Handbook of the Neuropsychology of Language, ed Faust M. (Chichester: Wiley-Blackwell), 368–385.

[B18] Cacciari C. Tabossi P. (1988). The comprehension of idioms. J. Mem. Lang. 27, 668–683. 10.1016/0749-596X(88)90014-9

[B19] Cardillo E. R. Watson C. E. Schmidt G. L. Kranjec A. Chatterjee A. (2012). From novel to familiar: tuning the brain for metaphors. Neuroimage 59, 3212–3221. 10.1016/j.neuroimage.2011.11.079 22155328 PMC3288556

[B20] Chakrabarty M. Sarkar S. Chatterjee A. Ghosal M. Guha P. Deogaonkar M. (2014). Metaphor comprehension deficit in schizophrenia with reference to the hypothesis of abnormal lateralization and right hemisphere dysfunction. Lang. Sci. 44, 1–14. 10.1016/j.langsci.2014.01.002

[B21] Chan R. C. K. Di X. McAlonan G. M. Gong Q. Y. (2011). Brain anatomical abnormalities in high-risk individuals, first-episode, and chronic schizophrenia: an activation likelihood estimation meta-analysis of illness progression. Schizophr. Bull. 37, 177–188. 10.1093/schbul/sbp073 19633214 PMC3004195

[B22] Chapman L. J. (1960). Confusion of figurative and literal usages of words by schizophrenics and brain damaged patients. J. Abnorm. Soc. Psychol. 60, 412–416. 10.1037/h0043371 13809245

[B23] Cohen J. (1988). Statistical Power Analysis for the Behavioral Sciences. Hillsdale, NJ: Lawrence Earlbaum Associates.

[B24] Colston H. L. Katz A. N. (2013). Figurative Language Comprehension: Social and Cultural Influences. New York, NY; Hove: Psychology Press.

[B25] Coulson S. Van Petten C. (2002). Conceptual integration and metaphor: an event-related potential study. Mem. Cognit. 30, 958–968. 10.3758/BF03195780 12450098

[B26] Coulson S. Federmeier K. D. Van Petten C. Kutas M. (2005). Right hemisphere sensitivity to word- and sentence-level context: evidence from event-related brain potentials. J. Exp. Psychol. Learn. Mem. Cogn. 31, 129–147. 10.1037/0278-7393.31.1.129 15641911

[B27] Crow T. J. (2000). Schizophrenia as the price that homo sapiens pays for language: a resolution of the central paradox in the origin of the species. Brain Res. Brain Res. Rev. 31, 118–129. 10.1016/S0165-0173(99)00029-6 10719140

[B28] de Bonis M. Epelbaum C. Deffez V. Feline A. (1997). The comprehension of metaphors in schizophrenia. Psychopathology 30, 149–154. 10.1159/000285041 9186980

[B29] DeLisi L. E. (2001). Speech disorder in schizophrenia: review of the literature and exploration of its relation to the uniquely human capacity for language. Schizophr. Bull. 27, 481–496. 10.1093/oxfordjournals.schbul.a006889 11596849

[B30] Denke C. Rotte M. Heinze H.-J. Schaefer M. (2014). Lying and the subsequent desire for toothpaste: activity in the somatosensory cortex predicts embodiment of the moral-purity metaphor. Cereb. Cortex 26, 477–484. 10.1093/cercor/bhu170 25214311

[B31] Desai R. H. Binder J. R. Conant L. L. Mano Q. R. Seidenberg M. S. (2011). The neural career of sensory-motor metaphors. J. Cogn. Neurosci. 23, 2376–2386. 10.1162/jocn.2010.21596 21126156 PMC3131459

[B32] Drury V. M. Robinson E. J. Birchwood M. (1998). 'Theory of mind' skills during an acute episode of psychosis and following recovery. Psychol. Med. 28, 1101–1112. 10.1017/S0033291798006850 9794017

[B33] Elmore C. M. Gorham D. R. (1957). Measuring the impairment of the abstracting function with the proverbs test. J. Clin. Psychol. 13, 263–266. 10.1002/1097-4679(195707)13:3<263::AID-JCLP2270130308>3.0.CO;2-C 13439039

[B34] Epelbaum C. Debonis M. Gineste M. D. (1992). Processing of mteaphoric expressions in schizophrenia - pilot studies. Eur. Rev. Appl. Psychol. 42, 117–128.

[B35] Ettinger U. Mohr C. Gooding D. C. Cohen A. S. Rapp A. Haenschel C.. (2015). Cognition and brain function in schizotypy: a selective review. Schizophr. Bull. 41(Suppl. 2), S417–S426. 10.1093/schbul/sbu190 25810056 PMC4373634

[B36] Faust M. Weisper S. (2000). Understanding metaphoric sentences in the two cerebral hemispheres. Brain Cogn. 43, 186–191. 10857691

[B37] Finckh J. (1906). Zur Frage der Intelligenzprüfung. Zentralblatt für Nerv. Psychiatr. 29, 945–957.

[B38] Fleischhacker H. (1930). Über Störungen des Sprachverständnisses bei Schizophrenen. Eur. Neurol. Monatsschrift für Psychiatr. Neurol. 77, 17–37. 10.1159/000164279

[B39] Gagnon L. Goulet P. Giroux F. Joanette Y. (2003). Processing of metaphoric and non-metaphoric alternative meanings of words after right- and left-hemispheric lesion. Brain Lang. 87, 217–226. 10.1016/S0093-934X(03)00057-9 14585291

[B40] Gibbs R. W. Jr. Colston H. L. (2012). Interpreting Figurative Meaning. Cambridge, UK:Cambridge University Press.

[B41] Gibbs R. W. Lima P. L. C. Francozo E. (2004). Metaphor is grounded in embodied experience. J. Pragmat. 36, 1189–1210. 10.1016/j.pragma.2003.10.009

[B42] Giora R. (1999). On the priority of salient meanings: studies of literal and figurative language. J. Pragmat. 31, 919–929. 10.1016/S0378-2166(98)00100-3

[B43] Giora R. (2007). Is metaphor special? Brain Lang. 100, 111–114. 10.1016/j.bandl.2006.08.001 16956657

[B44] Giora R. Fein O. (1999). On understanding familiar and less-familiar figurative language. J. Pragmat. 31, 1601–1618. 10.1016/S0378-2166(99)00006-5

[B45] Glucksberg S. (2003). The psycholinguistics of metaphor. Trends Cogn. Sci. 7, 92–96. 10.1016/S1364-6613(02)00040-2 12584028

[B46] Goldstein K. (1939). The significance of special mental tests for diagnosis and prognosis in schizophrenia. Am. J. Psychiatry. 96, 575–588. 10.1176/ajp.96.3.575

[B47] Gorham D. R. (1956). Use of the proverbs test for differentiating schizophrenics from normals. J. Consult. Psychol. 20, 435–440. 10.1037/h0042949 13376774

[B48] Gutmann M. L. (2009). The Effect of Frontal Lobe Function on Proverb Interpretation in Parkinson's Disease. ProQuest; The University of Arizona.

[B49] Hadlich H. (1931). Schizophrene Denkstörung. Psychol. Res. 15, 359–373. 10.1007/BF00406047

[B50] Heckers S. (1997). Neuropathology of schizophrenia. Schizophr. Bull. 23, 403–421. 10.1093/schbul/23.3.403 9327506

[B51] Hensler M. M. (2009). Sind Konkretistische Denkstörungen Eine Homogene Entität? Untersuchungen zum Verständnis Nicht-Wörtlicher Sprache Bei Schizophrenen Patienten. Thesis, Medical faculty, University of Tübingen, Germany Available online at: http://hdl.handle.net/10900/45461

[B52] Herold R. Tenyi T. Lenard K. Trixler M. (2002). Theory of mind deficit in people with schizophrenia during remission. Psychol. Med. 32, 1125–1129. 10.1017/S0033291702005433 12214792

[B53] Humphrey M. K. Bryson F. M. Grimshaw G. M. (2010). Metaphor processing in high and low schizotypal individuals. Psychiatry Res. 178, 290–294. 10.1016/j.psychres.2009.06.002 20493534

[B54] Iakimova G. Passerieux C. Hardy-Bayle M. C. (2006). The understanding of metaphors in schizophrenia and depression. An experimental approach. Encephale 32, 995–1002. 10.1016/S0013-7006(06)76279-0 17372545

[B55] Jäger A. O. Althoff K. (1994). Der WILDE-Intelligenz-Test:(WIT) ein Strukturdiagnostikum. Göttingen: Hogrefe; Verlag für Psychologie.

[B56] Kanner L. (1946). Irrelevant and metaphorical language in early infantile autism. Am. J. Psychiatry 103, 242–246. 10.1176/ajp.103.2.242 21001998

[B57] Kasanin J. S. (1944). Language and Thought in Schizophrenia. Berkeley: University of California Press.

[B58] Kircher T. (2003). Neuronale Korrelate Psychopathologischer Symptome. Denk-und Sprachprozesse bei Gesunden und Patienten mit Schizophrenie. Darmstadt: Steinkopff.

[B59] Kircher T. T. Bulimore E. T. Brammer M. J. Williams S. C. Broome M. R. Murray R. M.. (2001). Differential activation of temporal cortex during sentence completion in schizophrenic patients with and without formal thought disorder. Schizophr. Res. 50, 27–40. 10.1016/S0920-9964(00)00042-6 11378312

[B60] Kircher T. T. Leube D. T. Erb M. Grodd W. Rapp A. M. (2007). Neural correlates of metaphor processing in schizophrenia. Neuroimage 34, 281–289. 10.1016/j.neuroimage.2006.08.044 17081771

[B61] Kleist K. (1914). Aphasie und Geisteskrankheit. Münchener Medizinische Wochenschrift 61, 8–12.

[B62] Kogan N. Chadrow M. (1986). Children's comprehension of metaphor in the pictorial and verbal modality. Int. J. Behav. Dev. 9, 285–295. 10.1177/016502548600900302

[B63] Krystal J. H. Karper L. P. Bennett A. D'Souza D. C. Abi-Dargham A. Morrissey K.. (1998). Interactive effects of subanesthetic ketamine and subhypnotic lorazepam in humans. Psychopharmacology 135, 213–229. 10.1007/s002130050503 9498724

[B64] Lai V. T. van Dam W. Conant L. L. Binder J. R. Desai R. H. (2015). Familiarity differentially affects right hemisphere contributions to processing metaphors and literals. Front. Hum. Neurosci. 9:44. 10.3389/fnhum.2015.00044 25713522 PMC4322727

[B65] Landau M. J. Meier B. P. Keefer L. A. (2010). A metaphor-enriched social cognition. Psychol. Bull. 136, 1045. 10.1037/a0020970 20822208

[B66] Langdon R. Coltheart M. (2004). Recognition of metaphor and irony in young adults: the impact of schizotypal personality traits. Psychiatry Res. 125, 9–20. 10.1016/j.psychres.2003.10.005 14967548

[B67] Langdon R. Coltheart M. Ward P. B. Catts S. V. (2002). Disturbed communication in schizophrenia: the role of poor pragmatics and poor mind-reading. Psychol. Med. 32, 1273–1284. 10.1017/S0033291702006396 12420896

[B68] Levy R. (1968). The effect of chlorpromazine on sentence structure of schizophrenic patients. Psychopharmacology 13, 426–432. 10.1007/BF00404958 4884171

[B69] Li X. Branch C. A. DeLisi L. E. (2009). Language pathway abnormalities in schizophrenia: a review of fMRI and other imaging studies. Curr. Opin. Psychiatry 22, 131–139. 10.1097/YCO.0b013e328324bc43 19553866

[B70] Martin I. McDonald S. (2005). Exploring the causes of pragmatic language deficits following traumatic brain injury. Aphasiology 19, 712–730. 10.1080/02687030500172203

[B71] Mashal N. Faust M. (2010). The effects of metaphoricity and presentation style on brain activation during text comprehension. Metaphor Symbol 25, 19–33. 10.1080/10926480903538464

[B72] Mashal N. Faust M. Hendler T. (2005). The role of the right hemisphere in processing nonsalient metaphorical meanings: application of principal components analysis to fMRI data. Neuropsychologia 43, 2084–2100. 10.1016/j.neuropsychologia.2005.03.019 16243053

[B73] Mashal N. Vishne T. Laor N. (2014). The role of the precuneus in metaphor comprehension: evidence from an fMRI study in people with schizophrenia and healthy participants. Front. Hum. Neurosci. 8:818. 10.3389/fnhum.2014.00818 25360101 PMC4199320

[B74] Mashal N. Vishne T. Laor N. Titone D. (2013). Enhanced left frontal involvement during novel metaphor comprehension in schizophrenia: evidence from functional neuroimaging. Brain Lang. 124, 66–74. 10.1016/j.bandl.2012.11.012 23291493

[B75] Mitchell R. L. Crow T. J. (2005). Right hemisphere language functions and schizophrenia: the forgotten hemisphere? Brain 128, 963–978. 10.1093/brain/awh466 15743870

[B76] Mo S. Su Y. Chan R. C. Liu J. (2008). Comprehension of metaphor and irony in schizophrenia during remission: the role of theory of mind and IQ. Psychiatry Res. 157, 21–29. 10.1016/j.psychres.2006.04.002 17854910

[B77] Moro A. Bambini V. Bosia M. Anselmetti S. Riccaboni R. Cappa S. F.. (2015). Detecting syntactic and semantic anomalies in schizophrenia. Neuropsychologia 79, 147–157. 10.1016/j.neuropsychologia.2015.10.030 26519554

[B78] Mossaheb N. Aschauer H. N. Stoettner S. Schmoeger M. Pils N. Raab M.. (2014). Comprehension of metaphors in patients with schizophrenia-spectrum disorders. Compr. Psychiatry 55, 928–937. 10.1016/j.comppsych.2013.12.021 24556517

[B79] Nunn J. Peters E. (2001). Schizotypy and patterns of lateral asymmetry on hemisphere-specific language tasks. Psychiatry Res. 103, 179–192. 10.1016/S0165-1781(01)00273-6 11549406

[B80] Papagno C. Caporali A. (2006). Testing idiom comprehension in aphasic patients: the effects of task and idiom type. Brain Lang. 100, 208–220. 10.1016/j.bandl.2006.01.002 16487581

[B81] Raposo A. Moss H. E. Stamatakis E. A. Tyler L. K. (2009). Modulation of motor and premotor cortices by actions, action words and action sentences. Neuropsychologia 47, 388–396. 10.1016/j.neuropsychologia.2008.09.017 18930749

[B82] Rapp A. (2009). The role of the right hemisphere for language in schizophrenia, in Language Lateralization and Psychosis, ed Sommer I. K. (Cambridge; New York, NY; Melbourne, VIC; Madrid; Cape Town; Singapore; Sao Paulo: Cambridge University Press), 147–156.

[B83] Rapp A. M. Steinhäuser A. E. (2013). Functional MRI of sentence-level language comprehension in schizophrenia: a coordinate-based analysis. Schizophr. Res. 150, 107–113. 10.1016/j.schres.2013.07.019 23911258

[B84] Rapp A. M. Wild B. (2011). Nonliteral language in Alzheimer dementia: a review. J. Int. Neuropsychol. Soc. 17, 207–218. 10.1017/S1355617710001682 21241530

[B85] Rapp A. M. Bayer W. Längle G. (2007). Is language-related psychopathology a predictor of psychosocial functioning in schizophrenia? Results from a 4 year follow up study. Schizophr. Bull. 33:S602.

[B86] Rapp A. M. Erb M. Grodd W. Bartels M. Markert K. (2011). Neural correlates of metonymy resolution. Brain Lang. 119, 196–205. 10.1016/j.bandl.2011.07.004 21889196

[B87] Rapp A. M. Langohr K. Mutschler D. E. Wild B. (2014). Irony and proverb comprehension in schizophrenia: do female patients “dislike” ironic remarks? Schizophr. Res. Treat. 2014:841086. 10.1155/2014/841086 24991434 PMC4060160

[B88] Rapp A. M. Langohr K. Mutschler D. E. Klingberg S. Wild B. Erb M. (2013). Isn't it ironic? Neural correlates of irony comprehension in schizophrenia. PLoS ONE 8:e74224. 10.1371/journal.pone.0074224 24040207 PMC3769349

[B89] Rapp A. M. Leube D. T. Erb M. Grodd W. Kircher T. T. (2004). Neural correlates of metaphor processing. Brain Res. Cogn. Brain Res. 20, 395–402. 10.1016/j.cogbrainres.2004.03.017 15268917

[B90] Rapp A. M. Mutschler D. E. Erb M. (2012). Where in the brain is nonliteral language? A coordinate-based meta-analysis of functional magnetic resonance imaging studies. Neuroimage 63, 600–610. 10.1016/j.neuroimage.2012.06.022 22759997

[B91] Rapp A. M. Mutschler D. E. Wild B. Erb M. Lengsfeld I. Saur R.. (2010). Neural correlates of irony comprehension: the role of schizotypal personality traits. Brain Lang. 113, 1–12. 10.1016/j.bandl.2009.11.007 20071019

[B92] Rapp A. Schmierer P. (2010). Proverbs and nonliteral language in Schizophrenia: a systematic methodological review of all studies published 1931–2010. Schizophr. Res. 117, 422 10.1016/j.schres.2010.02.775

[B93] Rhodes J. E. Jakes S. (2004). The contribution of metaphor and metonymy to delusions. Psychol. Psychother. 77, 1–17. 10.1348/147608304322874227 15025901

[B94] Schettino A. Lauro L. R. Crippa F. Anselmetti S. Cavallaro R. Papagno C. (2010). The comprehension of idiomatic expressions in schizophrenic patients. Neuropsychologia 48, 1032–1040. 10.1016/j.neuropsychologia.2009.11.030 19963000

[B95] Schneider S. Wagels L. Haeussinger F. B. Fallgatter A. J. Ehlis A. C. Rapp A. M. (2015). Haemodynamic and electrophysiological markers of pragmatic language comprehension in schizophrenia. World J. Biol. Psychiatry 16, 398–410. 10.3109/15622975.2015.1019359 25816925

[B96] Schnell Z. (2007). Metaphor processing and the acquisition of idioms: a mentalistic model. Acta Linguist. Hung. 54, 73–104. 10.1556/ALing.54.2007.1.3

[B97] Schonauer K. Buchkremer G. (1986). Zur sprachlichen manifestation schizophrenen denkens außerhalb akuter krankheitsepisoden. Eur. Arch. Psychiatry Neurol. Sci. 236, 179–186. 10.1007/BF00380947 3803402

[B98] Sela T. Lavidor M. Mitchell R. L. C. (2015). A possible contributory mechanism for impaired idiom perception in schizophrenia. Psychiatry Res. 229, 1–11. 10.1016/j.psychres.2015.07.021 26216166

[B99] Sparks A. McDonald S. Lino B. O'Donnelle M. Green M. J. (2010). Social cognition, empathy and functional outcome in schizophrenia. Schizophr. Res. 122, 172–178. 10.1016/j.schres.2010.06.011 20609567

[B100] Spitzer M. Braun U. Hermle L. Maier S. (1993). Asssociative semantic network dysfunction in thought-disordered schizophrenic patients - direct evidence from indirect semantic priming. Biol. Psychiatry 34, 864–877. 10.1016/0006-3223(93)90054-H 8110913

[B101] Spitzer M. Lukas M. Maier S. Hermle L. (1994). Das verstehen metaphorischer rede bei gesunden probanden und schizophrenen patienten. Ein experientalpsychopathologischer Beitrag zum Konkretismus. Nervenarzt 65, 282–292. 8052330

[B102] Storch A. (1922). Das archaisch-primitive Erleben und Denken der Schizophrenen, in Monographien aus Dem Gesamtgebiet der Neurologie und Psychiatrie, Vol. 32, eds Müller-Rüfenacht M. Spatz H. Vogel P. (Berlin; Heidelberg: Springer), 321–341.

[B103] Straube B. Green A. Sass K. Kircher T. (2014). Superior temporal sulcus disconnectivity during processing of metaphoric gestures in schizophrenia. Schizophr. Bull. 40, 936–944. 10.1093/schbul/sbt110 23956120 PMC4059440

[B104] Thoma P. Hennecke M. Mandok T. Waehner A. Bruene M. Juckel G.. (2009). Proverb comprehension impairments in schizophrenia are related to executive dysfunction. Psychiatry Res. 170, 132–139. 10.1016/j.psychres.2009.01.026 19906437

[B105] Titone D. Holzman P. S. Levy D. L. (2002). Idiom processing in schizophrenia: literal implausibility saves the day for idiom priming. J. Abnorm. Psychol. 111:313. 10.1037/0021-843X.111.2.313 12003452

[B106] Uekermann J. Thoma P. Daum I. (2008). Proverb interpretation changes in aging. Brain Cogn. 67, 51–57. 10.1016/j.bandc.2007.11.003 18164527

[B107] Varga E. Schnell Z. Tenyi T. Nemeth N. Simon M. Hajnal A. (2014). Compensatory effect of general cognitive skills on non-literal language processing in schizophrenia: a preliminary study. J. Neurolinguistics 29, 1–16. 10.1016/j.jneuroling.2014.01.001

[B108] Watson C. G. (1976). The relationships of the process/reactive, paranoid/nonparanoid, length of illness, and length of hospitalization dimensions to schizophrenic abstract thinking deficits. J. Nerv. Ment. Dis. 163, 334–340. 10.1097/00005053-197611000-00006 978189

[B109] Winner E. Gardner H. (1977). The comprehension of metaphor in brain-damaged patients. Brain 100, 717–729. 10.1093/brain/100.4.717 608117

[B110] Zeev-Wolf M. Faust M. Levkovitz Y. Harpaz Y. Goldstein A. (2015). Magnetoencephalographic evidence of early right hemisphere overactivation during metaphor comprehension in schizophrenia. Psychophysiology 52, 770–781. 10.1111/psyp.12408 25603893

[B111] Zeev-Wolf M. Goldstein A. Levkovitz Y. Faust M. (2014). Fine-coarse semantic processing in schizophrenia: a reversed pattern of hemispheric dominance. Neuropsychologia 56, 119–128. 10.1016/j.neuropsychologia.2014.01.008 24462952

